# Disseminated tuberculosis presenting with polymorphonuclear effusion and septic shock in an HIV-seropositive patient: a case report

**DOI:** 10.1186/1752-1947-4-155

**Published:** 2010-05-26

**Authors:** Olivier Nancoz, Omar Kherad, Etienne Perrin, Christophe Hsu, Johannes Alexander Lobrinus, Mathieu Nendaz

**Affiliations:** 1 Department of Internal Medicine, Geneva University Hospitals, Geneva, Switzerland; 2 Division of Pulmonary Diseases, Geneva University Hospital, Geneva, Switzerland; 3 Department of Pathology, Geneva University Hospitals, Geneva, Switzerland

## Abstract

**Introduction:**

Because a substantial number of patients present with few or atypical symptoms, the recognition of tuberculosis remains challenging. Disseminated tuberculosis presenting with septic shock has already been described in some case reports, but, to the best of our knowledge, it has never been associated with polymorphonuclear effusion.

**Case presentation:**

We describe the case of a 27-year-old man from western Africa who was seropositive for human immunodeficiency virus. He presented with pleural and abdominal polymorphonuclear effusions and quickly developed septic shock due to disseminated *Mycobacterium tuberculosis *infection leading to multiple organ failure and death.

**Conclusion:**

In high-risk patients, *Mycobacterium tuberculosis *infection should be considered even in exceptional clinical presentations, such as septic shock and polymorphonuclear effusions.

## Introduction

The prevalence of tuberculosis (TB) in developed countries has decreased since the 1990s, which reflects worldwide efforts to properly identify and treat TB according to World Health Organization (WHO) recommendations. Nevertheless, TB remains a leading problem in public health, notably because of the poor living conditions of some parts of the population (for example, immigrants from countries with a high prevalence of TB) and the incidence of patients who are seropositive for the human immunodeficiency virus (HIV). In Geneva, Switzerland, the incidence of TB is 20 per 100,000 for a population of 440,000 inhabitants.

Because a substantial number of patients present with few or atypical symptoms, which mostly, but not exclusively, present in immunocompromised patients, the recognition of TB remains challenging. The time between the presentation of symptoms and diagnosis may also turn out excessively long, with a median delay of 2.1 months in cases documented in Geneva, and even six months in extreme cases [1].

We describe the case of patient with HIV who presented with atypical polymorphonuclear effusions and quickly developed a septic shock due to disseminated TB.

## Case presentation

A 27-year-old man from western Africa without any relevant medical history presented to the emergency department of our hospital with a two-month history of cough, intermittent fever, weight loss of 12 kg and profuse diarrhea.

On examination, our patient appeared lean at a body mass index (BMI) of 19 kg/m^2^, with a pulse rate of 116/minute, blood pressure of 130/90 mmHg, breathing rate of 25/minute, and temperature of 37.6°C. Results of his cardiovascular examination were normal. His chest examination revealed hypoventilation and dullness on both pulmonary bases. His abdomen was distended and diffusely tender, with posterior dullness. A psychomotor agitation with mild confusion was present, without neurological deficit.

Results of his blood tests are shown in Table 1. His HIV serology test (MEIA^®^, COMBO^® ^and Immunoblot^®^) returned positive. Samples of his blood and urine were sent for typical bacteriology cultures and returned negative (after 48 hours for urine and six days for blood). His chest radiography showed bilateral pleural effusion. An abdominal ultrasound of our patient revealed the presence of peritoneal fluid and hepatosplenomegaly, with parenchymatous hypoechogenic lesions in both organs and nodular retroperitoneal images.

**Table 1:** Blood test result.

	Patient value	Normal value
Hemoglobin	125 g/L	140-180 g/L
White cell	6.6 G/l	4-11 G/L
Non-segmented neutrophils	18%	0-5%
Platelet	99 G/L	150-350 G/L
C-reactive protein	298 mg/L	0-10 mg/L
Creatinin	270 μmol/L	62-106 μmol/L
Urea (BUN)	24.7 mmol/L	2.6-7.1 mmol/L
ASAT	198 U/L	14-50 U/L
ALAT	48 U/L	12-50 U/L
Alkalin phosphatase	220 U/L	30-125 U/L
Lactate dehydrogenase	1945 U/L	125-240 U/L
Total bilirubin	38 μmol/L	7-25 μmol/L
Conjugated bilirubin	16 μmol/L	2-9 μmol/L
Prothrombin time	85%	80-120%
Partial thromboplastin time	29.6 sec	25-32 sec

ALAT: alanine transaminase; ASAT: aspartate aminotransferase; BUN: blood urea nitrogen.

An analysis of our patient's pleural effusion showed an exudative fluid with the following values: lactate dehydrogenase (LDH) 1668 U/L, proteins 57 g/L, and glucose 6.4 mmol/L. There were 1932 leukocytes/mm^3 ^with 66% neutrophils, 22% lymphocytes, 9% plasmocytes, 1% macrophages, 2% mesothelial cells, and 0% eosinophils. His ascites analysis revealed 3060 leukocytes/mm^3 ^with 64% neutrophils, 18% lymphocytes, 15% plasmocytes, and 3% macrophages. His Gram, acridine and auramine stains were negative on both fluids upon direct examination. No acid-fast bacilli could be detected by direct examination of his sputum.

**Figure 1:**
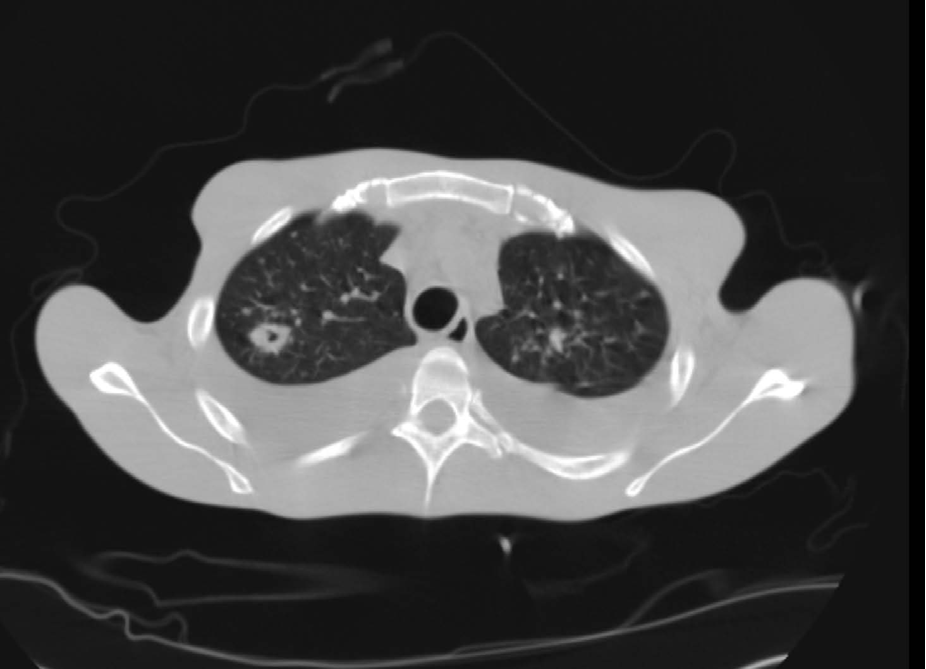
**Computed tomography of the chest with bilateral pleural effusion and multiple nodular lesions, one of which is excavated**.

Results of our patient's transthoracic cardiac echography were normal, except for a moderate, inhomogeneous impairment of his left ventricular ejection fraction (35% to 40%), with global hypokinesy involving the middle part of his left ventricle, septum and apex. A native thoracoabdominal computed tomography confirmed abdominal and pleural fluid effusions and showed multiple pulmonary and splenic nodules. It also showed diffuse mesenteric and para-aortic adenopathies. Our patient's thoracic scan is shown on Figure 1.

We then started our patient on an empirical treatment of imipenem and cilastine, which was completed by a standard antituberculous quadritherapy of rifampicin, isoniazid, pyrazinamide, and ethambutol due to high TB suspicion.

Our patient subsequently developed a rapidly progressive septic shock and died 24 hours later despite attempts at resuscitation.

**Figure 2:**
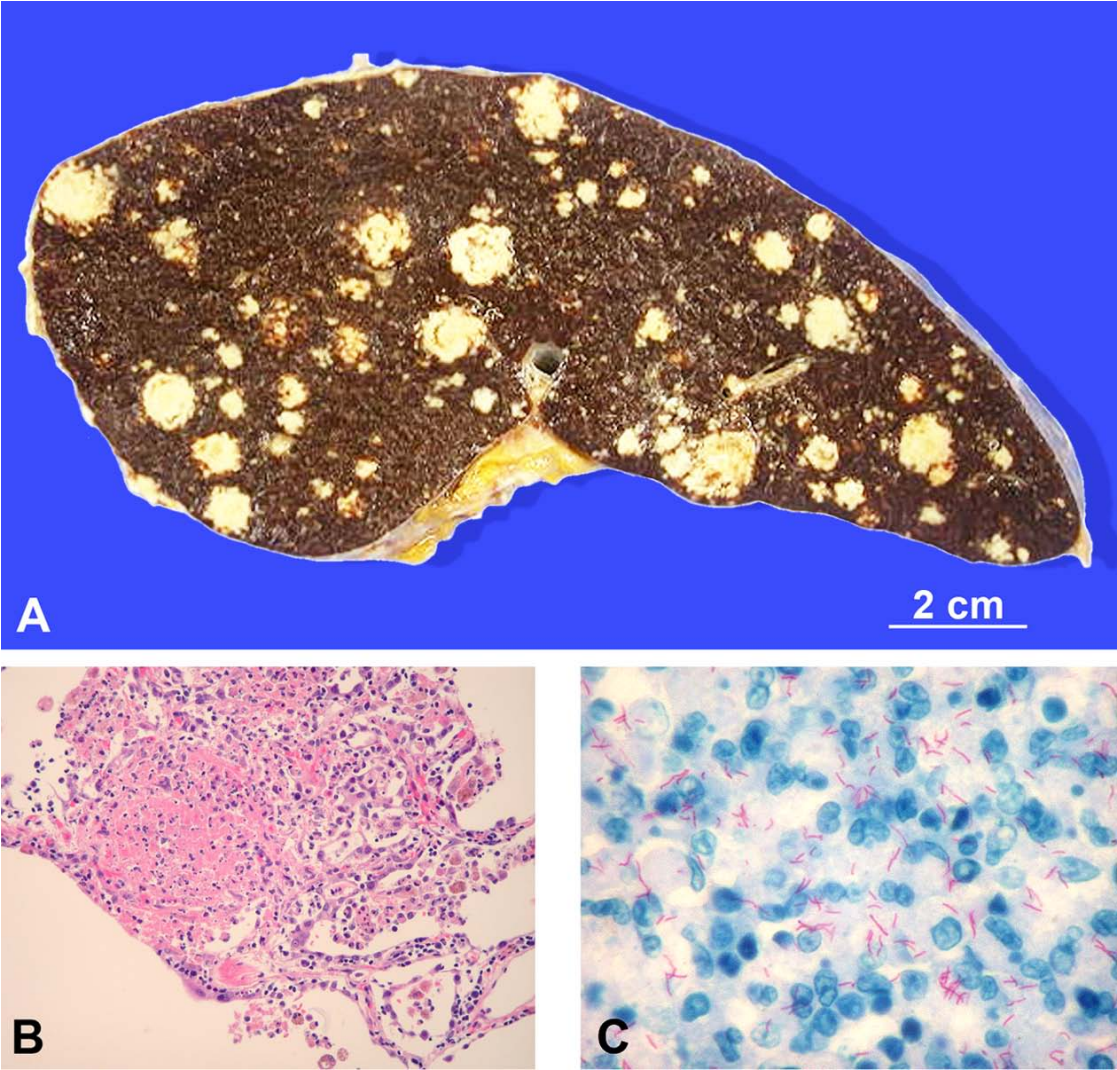
**(A) Macroscopic transverse section of enlarged spleen with multiple white creamy nodules**. **(B) **Microscopic view of a pulmonary granuloma (hematoxylin and eosin stain, magnification 200×). **(C) **High magnification microscopic view showing numerous acid-fast bacilli (Ziehl-Neelsen stain, magnification 600×).

An isoniazid-resistant strain of *Mycobacterium tuberculosis *was cultured from our patient's pleural and abdominal effusions, as well as from his urine and post-mortem bronchial aspirations. No other bacteria were identified. A post-mortem examination performed less than three hours after his death showed bilateral pleural effusion (1100 cc on the left and 1220 cc on the right side) and ascites (2500 cc). Multiple nodules between 1 mm and 15 mm in size were also observed in his lungs, pleura, pericardium, liver (2250 g), spleen (490 g, see Figure 2A), peritoneum and omentum, pancreas, adrenal glands, thoracic, abdominal and retroperitoneal lymph nodes, and bone marrow.

Histologically, the nodules corresponded to necrotizing granulomas, with very abundant polymorphonuclears (see Figure 2B). Ziehl-Neelsen staining of these nodules revealed numerous acid-fast bacteria (see Figure 2C), while Gram and silver staining did not show any other bacteria, fungus or parasite. The post-mortem culture of our patient's tracheal aspirate, lung tissue and omentum returned positive for *M. tuberculosis*. No histological signs of cytomegalovirus or herpetic infections were present. Apart from esophageal candidiasis, no other pathological conditions associated with HIV, such as *Pneumocystis jirovecii *infection, cerebral toxoplasmosis or lymphoma, Kaposi sarcoma, or HIV-related lymphadenopathy, were found.

## Discussion

Despite rapid administration of anti-tuberculosis drugs after admission, our patient developed a devastating septic shock with multiple organ failure, diffuse effusions, and multiple polymorphonuclear rich necrotizing granulomas infiltration of all tissues due to a *M. tuberculosis *bacteremia. Of particular interest in this case is the recognition of potentially misleading features, such as the presentation of symptoms with a quickly evolving septic shock, and the presence of polymorphonuclear effusions.

Although most cases of sepsis syndrome have a bacterial or toxic cause, TB presenting with septic shock in patients with HIV has already been recognized [2-5]. However, to the best of our knowledge, none of these patients had a polymorphonuclear effusion upon diagnosis. Moreover, there is limited information about the epidemiological characteristics of patients who are HIV-negative with *M. tuberculosis *septic shock. A recent study summarized the demographic and clinical characteristics of 27 cases of TB bacteremia in non-HIV patients reported in the literature [6]. Some case reports describe miliary tuberculosis, acute empyema, or sepsis and multi-organ failure [6-9], but, to the best of our knowledge, the presence of all these conditions in a single patient has never been documented.

Polymorphonuclear effusions (>60% PMN) is an atypical and rare hallmark of this tuberculosis case [10]. Although pleural effusion (*pleuritis exudativa tuberculosa*) is a common presentation of TB, it consists of a serous exudate with a nucleated cell count typically showing more than 85% to 90% lymphocytes [11], which are interpreted as a delayed hypersensitivity reaction rather than a direct tuberculous infection [12]. Conversely, rich polymorphonuclear pleural effusions can be seen in acute or early forms of direct pleural tuberculous dissemination (up to the first two weeks) [13] due to a rupture from a sub-pleural caseous focus, a rupture of a cavitation in the pleural space, a direct hematogenous spread, or a contamination by adjacent infected lymph nodes or a sub-diaphragmatic process [9,14]. Such events are more frequently documented in patients with TB parenchymatous infection.

The direct examination of cultures and early pleural fluid by Ziehl-Neelsen staining are often insufficient to confirm the appropriate diagnosis. Indeed, less than 5% to 10% percent (20% by patients with HIV) of pleural fluid staining register positive for acid-fast bacilli. Moreover, cultures return positive in 24% to 58%, with the majority of series showing less than 30% [14], and is limited by the long delay in obtaining results. The pleural biopsy for combined histological examination and culture is the most sensitive diagnostic method, but may still be falsely negative in 15% to 20% of documented cases [15].

## Conclusions

Although septic shock and polymorphonuclear pleural effusions have both been reported as atypical and rare presentations of tuberculous infection, the association of these two features makes the situation of our patient even more unusual. To the best of our knowledge, this association has not been previously reported and may represent a potential diagnostic pitfall. In high-risk patients, *M. tuberculosis *infection should be considered even in exceptional clinical presentation, such as septic shock and polymorphonuclear effusions. The case of our patient also illustrates the dramatic consequences of some forms of this disease, as well as the necessity to initiate anti-TB drugs quickly, pending confirmation by culture.

## Consent

Written informed consent could not be obtained from the patient for publication of this because the patient is now deceased and we were unable to contact a next-of-kin despite reasonable attempts. Every possible effort has been made to conceal the identity of the patient and we believe that a reasonable family would not object to publication of this case report.

## Competing interests

The authors declare that they have no competing interests.

## Authors' contributions

ON analyzed and interpreted our patient data and was a major contributor in writing the manuscript. OK analyzed and interpreted our patient data and was a major contributor in writing the manuscript. EP analyzed the data and was involved in drafting the manuscript and revising it critically. CH and JAL performed the autopsy and the histological examination of our patient's spleen and lungs, and contributed in writing the manuscript. MN analyzed the data and was involved in drafting the manuscript and revising it critically. All authors read and approved the final manuscript.
